# Gate voltage and doping effects on near-field radiation heat transfer in plasmonic heterogeneous pairs of graphene and black phosphorene†

**DOI:** 10.1039/c9ra04695j

**Published:** 2019-09-17

**Authors:** Desalegn T. Debu, M. Hasan Doha, Hugh O. H. Churchill, Joseph B. Herzog

**Affiliations:** Department of Physics, University of Arkansas Fayetteville Arkansas 72701 USA debudt@udel.edu; Institute of Nanoscale Science and Engineering, University of Arkansas Fayetteville Arkansas 72701 USA; R. B. Annis School of Engineering, University of Indianapolis Indianapolis IN USA 46227

## Abstract

Plasmon coupling and hybridization in 2D materials plays a significant role for controlling light–matter interaction at the nanoscale. We present a near-field radiation heat transfer (NFRHT) between vertically separated graphene and black phosphorene sheets at different temperatures in nanoscale separations. Radiation exchange from the theory of fluctuation electrodynamics is modulated by the carrier density of graphene and phosphorene. Direct comparison of NFRHT black phosphorene–graphene to symmetric graphene–graphene radiation exchange can be as much as 4 times higher for the selected doping range in both armchair (AC) and zigzag (ZZ) orientations of BP. The strong NFRHT enhancement of the specific optical properties of the heterogenous 2D material is due to the strong coupling of propagating surface plasmon polaritons as demonstrated by the distribution of the heat transfer coefficient. We also demonstrate that the magnitude of the near-field radiation enhancement is found to acutely depend on the vacuum gap of the graphene and BP pair. Interestingly, for separation distances below 200 nm, the total near-field heat transfer between black phosphorene and graphene exceeds that between graphene and graphene by 5 times. The radiation enhancement can be further tuned based on the orientation, AC, and ZZ of black phosphorene. These results prominently enable dynamic control of the total NFRHT relying on tunable anisotropic characteristics of BP irrespective of graphene's optical conductivity. Furthermore, the heterogeneous pairs of 2D materials potentially provide alternative platforms to achieve beyond super-Planckian radiation.

## Abstract 2

Plasmon coupling and hybridization in 2D materials plays a significant role for controlling light–matter interaction at the nanoscale.

## Graphical abstract

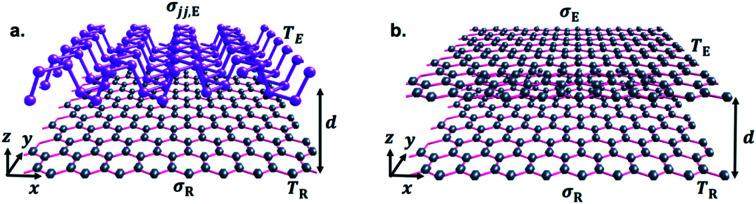

## Introduction

Near-field radiative heat transfer (NFRHT) between two bodies separated by a distance much smaller than or comparable to the thermal wavelength can break the blackbody (BB) limit.^1,2^ In-depth understanding of this phenomenon is not only essential for fundamental scientific importance but also offers a wide range of potential applications such thermal nanoimaging,^3^ near-field thermophotovoltaics,^4^ and thermal rectifiers.^5^ The NFRHT enhancement by several orders of magnitude has been widely investigated in materials that support phonon polaritons,^6^ resonant surface phonon polaritons (SphPs),^7^ surface plasmon polaritons (SPPs)^8^ or hyperbolic phonon polariton (HPP) modes^9,10^ due to evanescent waves. Control of NFRHT has been covered in several structural arrangements and materials;^11–17^ however, a challenge arises in enhancing NFRHT due to the large number of independent parameters, and more importantly, lack of degree of freedom for active tunability.

In this context, a breakthrough for active tuning mechanics has been effectively achieved in two-dimensional (2D) semiconductors: prominently graphene or MoS_2_ and, very recently, black phosphorene.^18–21^ Graphene exhibits highly tunable optical properties *via* voltage-gating,^18,22^ good thermal stability,^23^ and high carrier mobility.^18^ Moreover, graphene supports strongly confined surface plasmons with a frequency that varies with the wave number.^24–26^ Theoretically predicted tuning and coupling of surface mode plasmon of a graphene sheet with a nearby body, resulting in a significant increase of the NFRHT,^27–30^ has been confirmed experimentally.^31^ In addition, the near-field active thermal radiation response of graphene in subwavelength geometries, graphene ribbon,^32,33^ periodic nanoribbon,^34^ or nanodisks^35–37^ has demonstrated enhanced radiation. Most recently, Black phosphorus (BP) is also reported to have a layer dependent tunable direct bandgap,^38^ thermal stability,^39^ high carrier mobility,^20,21^ and remarkable in-plane anisotropic electrical and optical properties.^40^ In contrast to graphene, BP shows polarization dependency in the anisotropic surface plasmon response due to an order of magnitude difference in the in-plane effective electron masses along the two crystal axes results.^41,42^ These plasmon properties allow for the realization of novel 2D polarization dependent and tunable NFRHT.^33,43–45^ Another interesting approach to study is the NFRHT between heterogenous 2D material, shown to be possible using a graphene sheet and a MoS_2_ sheet.^46^ However, both graphene and MoS_2_ are nearly isotropic materials. This makes the graphene and MoS_2_ orientation indiscriminate in light matter interaction. Recently, the NFRHT of the graphene (GR) heterogenous (GR–BP) in contact with bulk substrate SiC has shown anisotropic effects in near field radiation heat transfer.^47^ However, SiC supports hyperbolic phonon polariton modes in the midinfrared range. In hybrid structures composed of 2D material, graphene/SiC or BP/SiC, SPPs can couple with hyperbolic phonon polariton.^47^ The hybrid modes move from the substrate and introduce a change in the propagation, and evanescent waves either greatly improve or reduce the NFRHT by creating a new channel. Therefore, it is important to understand the NFRHT and tuning capability graphene and BP heterogeneous system without dispersive hyperbolic layer.

Through numerical and analytic solutions based on the transfer matrix method combined with fluctuation electrodynamics,^48^ the near-field radiation transfers energy between the BP and the graphene, which are compared to two identical suspended graphene sheets by analysing the distribution of the heat transfer coefficient of the emitter and the receiver 2D layers. The NFRHT model shows that BP–GR has a more selective optical modulation compared to the graphene–graphene system. Furthermore, the NFRHT differences between these two systems are explored, considering the inherent BP optical anisotropy nature. The effect of the reorientation of BP is found to be significant in modulating the NFRHT, perhaps creating possible conditions roughly equivalent to or greater than graphene. The hybrid modes, which evolve from low to high parallel wave vectors in the infrared range, controlled by graphene and BP optical parameters determine the tunnelling probability. We also discuss the total radiation, enhancement in the 2D structure relative to the value of separation distance, offering potential routes toward passive or active control of NFRHT. In particular, we examined the ratio of total heat transfer of BP–GR and GR–GR, and its dependence on the anisotropy, doping, and spacing.

## Theoretical model

### Optical properties of graphene and black phosphorene

The optical conductivity in graphene, *σ*_g_(*ω*), is modelled with a 2D sheet conductivity that includes the contribution of an intraband transition term *σ*_D_ (*i.e.*, Drude), and interband transitions term, *σ*_I_, where *σ*_g_(*ω*) = *σ*_D_(*ω*) + *σ*_I_(*ω*).^49^1
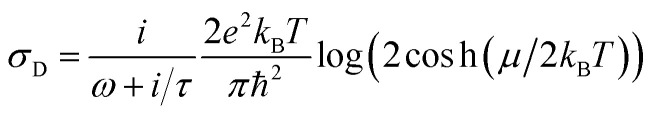
and2
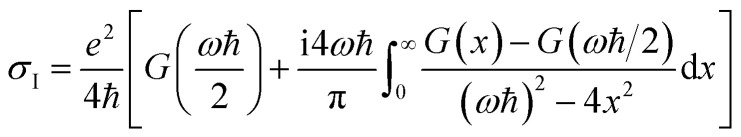
where *G*(*x*) = sinh[*x*/*k*_B_*T*]/[cosh(*μ*/*k*_B_*T*) + cosh(*x*/*k*_B_*T*)], *e* is the electron charge, *ℏ* is the reduced Planck constant, *k*_B_ is the Boltzmann constant, *μ* is the chemical potential, *T* is temperature, *ω* is the angular frequency, and *τ* is the relaxation scattering time, which we have fixed to the value *τ* = 10^−13^ s.^50^

The anisotropic layer dependent surface conductivity, *σ*_jj_, of BP is given by a simple semiclassical Drude model in the infrared regime,^40^3
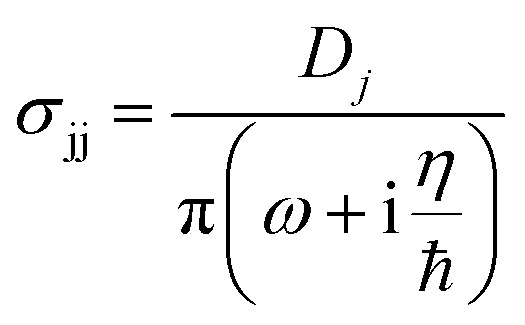
where *j* represents AC and ZZ-direction *η* describes the relaxation rate and is chosen to be 10 meV^40^ which is within the range obtained from ab-simulation^51^ and *D*_i_ = π*ne*^2^/*m*_*j*_ is the anisotropic Drude weight, *n* is the electron doping, and *m*_j_ represents the layer dependent effective mass of electrons along the j, (AC or ZZ) direction. In this work we use monolayer BP with arm-chair, *m*_*x*_ = 0.15*m*_0_, and zigzag, *m*_*y*_ = 0.7*m*_0_, direction masses, where *m*_0_ is the free electron mass.^40^

### Near-field radiation heat transfer

**Fig. 1:**
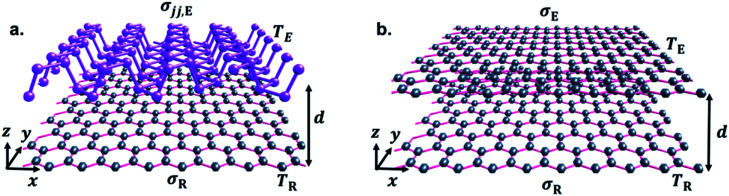
Schematic illustration of the emitter and receiver with temperatures *T*_E_ (hot) and *T*_R_ (cold) 2D material. The vacuum gap distance, *d*, and optical conductivity, *σ*, of the graphene sheet and the black phosphorene sheet are labeled. (a) Graphene sheet emitter (E) and black phosphorene sheet receiver (R), and (b) graphene sheet emitter and graphene sheet receiver.

Before setting the model of radiation heat transfer, we examined the light–matter interaction for the suspended isotropic 2D materials. As shown in Fig. 1, each two-dimensional layer material is represented by the conductivity tensor *σ*_ij_, consisting of transverse conductivities, longitudinal conductivities, and crossed conductivity components, which lies in the *xy*-plane. We obtain the Fresnel coefficients solving Maxwell's electromagnetic equation imposing the appropriate electromagnetic field boundary condition and representing the 2D conducting sheet surface current (*J*_s_) from Ohm's law. We assume that the electric field *E*_i_ in medium i, and *E*_j_ in medium j, i and j indicated medium above and below the 2D layer, with dielectric permittivity *ε*_i_ = *ε*_j_ = 1.0. The corresponding magnetic field components, *H*_i_ and *H*_j_, can be found using Maxwell's equation. The boundary conditions of the parallel and perpendicular gives *n̂* × (*E*_i_ − *E*_j_) = 0, *n̂* × (*H*_i_ − *H*_j_) = *J*_s_. The unit vector normal to the 2D layer is *n̂* = −*ẑ*, and *J*_s_ = *σ*_ij_*E*. Solving the boundary conditions, the Fresnel coefficients of reflection for s, p and the cross-polarization term expressions are^52,53^4
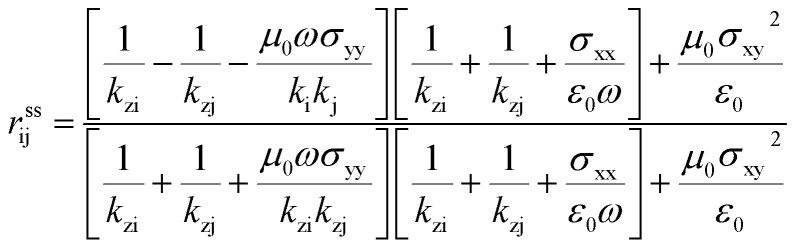
5
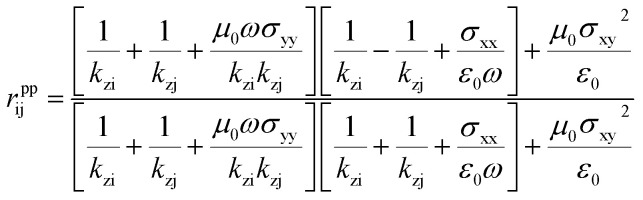
6
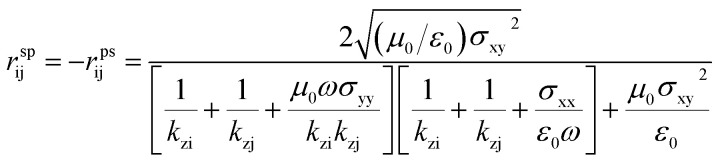
where i and j are the mediums that encapsulate graphene or BP. *r*^pp^_ij_, *r*^ss^_ij_ and *r*^sp^_ij_ are the Fresnel coefficients between the medium i and j for s, p and the mixture sp polarization. The z-component wavevector for s and p-polarization along each region is 
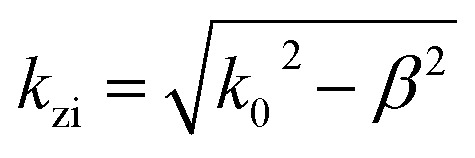
, 
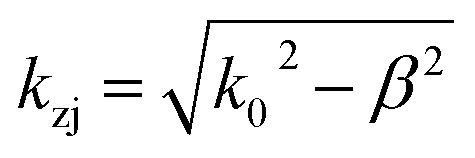
, and *ε*_0_ is the vacuum permittivity, *μ*_0_ is the vacuum permeability and *β* is the wave vector parallel to the surfaces of the 2D layers. This work studies BP AC orientation aligned with *x*-direction and ZZ along *y*-axis and *vice versa*. The incident polarization direction is collinear to one of the principal BP directions so that the crossed conductivity effect, *σ*_*xy*_ = 0, results in the cross-polarization *r*^ps^ = 0. A similar method has been followed recently by Xiao-Jie Yi *et al.*^47^ A rotated BP from the optical axis does change the momentum vector of along *x* and *y* and hence the expression of the reflection spectra.^54^

Now the NFRHT initiated by the coupling of the propagating surface modes meditated between the thermally hot (*T*_E_) and the thermally cold (*T*_R_) surfaces can be formulated following the framework of fluctuational electrodynamics as:^55^7
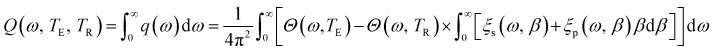
where *q*(*ω*) is the net spectral heat flux, *Θ*(*ω*,*T*) = *ωℏ*/(exp(*ωℏ*/*k*_B_*T*) − 1)is the average energy of Planck's oscillator, and *ξ*_s(p)_(*ω*,*β*) is the energy transmission coefficient that depends on the frequency and wave vector corresponding to s(p)-polarization of light. The energy transmission coefficient considering polarization coupling and contribution of evanescent modes, *β* > *k*_0_, and propagating modes, *β* < *k*_0_, is given by^56^8
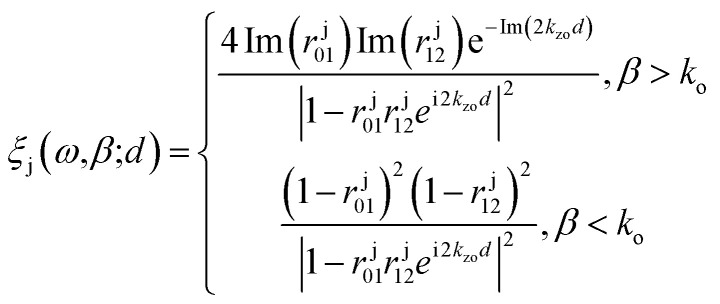
where j stands for either s or p polarization, 01 and 12 respectively denote the receiver and emitter medium reflection, *k*_0z_ is the normal wavevector component in vacuum of BP and graphene, Im() takes the imaginary part of a complex quantity, and *d* is the distance of the vacuum gap.

Throughout the paper, we consider the configuration in Fig. 1, where two 2D bodies with conductivity labelled as *σ*_jj,E_ or *σ*_E_ (emitter) and *σ*_R_ (receiver), respectively, are brought into proximity with a vacuum gap separation *d*. The temperatures of the emitter and the receiver are labeled *T*_E_ = 300 K and *T*_R_ = 290 K, (*T*_E_ > *T*_R_), respectively. We represent gate modulation or applied voltage bias effect in terms of changing the carrier density *n*, of graphene and BP. This indirect relation of gate-modulation of BP and graphene can be controlled independently leading to more degrees of freedom. The carrier density potential of graphene can be described also in terms of chemical potential, *n*_g_ = *μ*^2^/(*ℏv*_f_)^2^π, where *v*_F_ = 10^6^ ms^−1^ is the Fermi velocity. The values of *n*_g_ used in this work ranges from 0.74 × 10^12^ cm^−2^ to 4.7 × 10^13^ cm^−2^, with an equivalent range of *μ* = 0.1 eV to 0.9 eV, and *n*_b_ = 2.0 × 10^12^ cm^−2^ to 9.0 × 10^13^ cm^−2^ in order to get optimum NFRHT.

## Results and discussion

### Doping and gate modulation effect

**Fig. 2:**
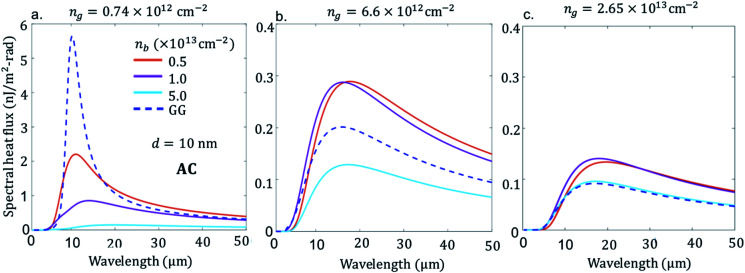
Spectral heat flux between suspended graphene sheet receiver (GRR), at *T*_R_ = 290 K, and black phosphorene sheet emitter (BPE) armchair (AC) direction along *x*-axis orientation, *T*_E_ = 300 K, separation distance, *d* = 10 nm, electron carrier density *n*_b_ = {5.0 × 10^12^ cm^−2^, 1.0 × 10^13^ cm^−2^, 5.0 × 10^13^ cm^−2^}, and carrier density of graphene (a) *n*_g_ = 0.74 × 10^12^ cm^−2^, (b) 6.6 × 10^12^ cm^−2^ and (c) 2.65 × 10^13^ cm^−2^. Blue dash line in all the figure is flux between two suspended graphene sheets at identical carrier density (a) *n*_gE_ = *n*_gR_ = *n*_g_ = 0.74 × 10^12^ cm^−2^, (b) 6.6 × 10^12^ cm^−2^ and (c) 2.65 × 10^13^ cm^−2^.

First, we consider the scenario in Fig. 2a–c, BP emitter (BPE) at temperature of 300 K and graphene receiver (GRR) at 290 K with the AC direction of BP aligned with *x*-direction and ZZ is in the *y*-direction. The separation distance *d* between graphene and BP is 10 nm. The figure shows the spectral heat flux (SHF) calculated from eqn (7) as a function of wavelength for GR low doped *n*_g_ = 0.74 × 10^12^ cm^−2^, 6.6 × 10^12^ cm^−2^ and high doped 2.65 × 10^13^ cm^−2^, which corresponds to a chemical potential of *μ* = 0.1 eV, 0.3 and 0.6 eV, respectively, and BP varying the doping-level, *n*_b_ = 0.5 × 10^13^ cm^−2^, 1.0 × 10^13^ cm^−2^, 5.0 × 10^12^ cm^−2^.

In all figures of Fig. 2a–c for comparison, we also included spectral heat flux (SHF) results calculated between two graphene sheets, graphene emitter (GRE)-graphene receiver (GRR) (blue broken line labelled as GG), see Fig. 1b. The same temperatures are used in all cases: *T*_E_ = 300 K and *T*_R_ = 290 K. We selected identical gate voltage for the emitter and receiver graphene, *n*_gE_ = *n*_gR_ = *n*_g_ since the latter case shows strong spectral heat flux.^38^ Therefore, low gate modulated NFRHT between identical graphene shows enhanced radiative energy transport than between low and highly gate modulated graphene. Further support of this conclusion is provided in the ESI Fig. 1 (a) and (b).† As seen in Fig. 2a, b and c, the SHF of identical graphene has higher *n*_g_ = 0.74 × 10^12^ cm^−2^ and decreases more than ten times when the doping is 2.65 × 10^13^ cm^−2^. In addition, at higher graphene carrier density, *n*_g_ = 2.65 × 10^13^ cm^−2^, SRH becomes broader due to weakening of interband effect.^27,29,38^ In the case of BPE–GRR, increasing number density *n*_b_ from 0.2 × 10^13^ cm^−2^ to 5.0 × 10^13^ cm^−2^ has a dramatic effect on the SHF at low carrier density, *n*_g_ = 0.74 × 10^12^ cm^−2^, Fig. 2a. The SHF peaks decrease gradually with the increase of number density by more than an order of magnitude. In addition, the SHF is broadening gradually increases and the peak value shifts slightly to a higher wavelength with increasing *n*. Compared to BPE–GRR for all *n*_g_ and *n*_b_, GRE–GRR is higher than BPE–GRR at *n*_g_ = 2.65 × 10^13^ cm^−2^. The role of the dependency of *n*_g_ and *n*_b_ on the SHF is further evaluated for 6.6 × 10^12^ cm^−2^ and 2.65 × 10^13^ cm^−2^, Fig. 2b and c. On the contrary, for *n*_g_ = 4 × 10^12^ cm^−2^ SHF for BPE–GRR is higher than the that of GRE–GRR from moderate to high doping, Fig. 2b, and for all doping level of BP, Fig. 2c. More specifically, for *n*_g_ = 6.6 × 10^12^ cm^−2^ the SHF of BPE–GRR is smaller than GRE–GRR and larger by a factor of two for *n*_b_ = 5.0 × 10^12^ cm^−2^ and 5.0 × 10^13^ cm^−2^. This indicates in BPE–GRR there is a critical *n*_b_ value between 5.0 × 10^12^ cm^−2^ and 1.0 × 10^13^ cm^−2^ making the transition of enhanced SHF than GRE–GRR, in Fig. 2b. For highly gated graphene, *n*_g_ = 2.65 × 10^13^ cm^−2^, we observe that BPE–GRR 2D system outperforms identical graphene sheets, within the whole range of *n*_b_, having two times higher SHF for *n*_g_ = 1.0 × 10^13^ cm^−2^ and 5.0 × 10^13^ cm^−2^.

**Fig. 3:**
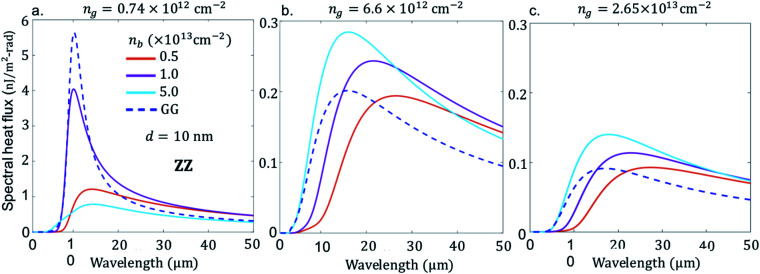
Spectral heat flux between suspended GRE–GRR and BPE–GRR, at *T*_E_ = 300 K, and black phosphorene sheet zigzag (ZZ) direction along *x*-axis orientation, *T*_R_ = 290 K, separation distance, *d* = 10 nm, electron carrier density *n*_b_ = 5.0 × 10^12^ cm^−2^, 1.0 × 10^13^ cm^−2^, 5.0 × 10^13^ cm^−2^, and blue dash line in all the figures is between two suspended graphene sheets at identical optical property *n*_g_ = 0.74 × 10^12^ cm^−2^ (a), *n*_g_ = 6.6 × 10^12^ cm^−2^ (b), and *n*_g_ = 2.65 × 10^13^ cm^−2^ (c).

So far, we have discussed the case of doping effect of BPE–GRR in AC orientation. However, besides electrostatic tuning of graphene and BP, effective mass of BP is readily different in the AC and ZZ direction, as shown in the optical conductivity eqn (2). In what follows, we rotate the BP arrangement as such the ZZ direction is along the *x*-axis and AC direction is along the *y*-axis. For this purpose, we show in Fig. 3 the dependence of the SHF (all parameters are same as in Fig. 2). The change in the orientation of the BP strongly modulates the height of the NFRHT spectra by the maximum peak energy and slightly changes the peak position. For example in Fig. 3a, the intermediate doping number density, when *n*_b_ is close to 1.0 × 10^13^ cm^−2^ for *n*_g_ = 0.74 × 10^12^ cm^−2^, reaches a minimum or a comparable density to GRE–GRR system. In the ZZ direction the SHF can be tuned by a factor of 2 to 4 between *n*_b_ = 0.5 × 10^13^ cm^−2^ and *n*_b_ = 1.0 × 10^13^ cm^−2^.

Noticeably at higher doping and lower doping, the SHF falls well below the GRE–GRR system. However, at both lower and higher doping levels, the NFRHT spectra of the BPE–GRR system does not reach the GRE–GRR value at 0.74 × 10^12^ cm^−2^, a consistent result of AC orientation (Fig. 2a). Likewise, as in the AC orientation case, the NFRHT of BPE–GRR is greater than the GRE–GRR system when *n*_g_ = 6.6 × 10^12^ cm^−2^ and *n*_g_ = 2.65 × 10^13^ cm^−2^ (Fig. 3a and b). To be more precise, when the doping level is bellow *n*_b_ = 0.5 × 10^13^ cm^−2^ we obtain a result less than the BPE–GRR, not shown in the figure. However, different from Fig. 3a, when *n*_g_ = 6.6 × 10^12^ cm^−2^ and *n*_g_ = 2.65 × 10^13^ cm^−2^, the SHF gradual spectra broadening and amplitude increase is also noticeable in the BPE–GRR system with an increase in *n*. This is due to the weakening of the interband loss in graphene. Both results in Fig. 2 and 3 indicate that irrespective of orientation and doping level of BP, the GRE–GRR system leads to higher radiation transfer than the BPE–GRR system for a small applied bias on the graphene. The gate modulation of graphene, anisotropic mass effect and the carrier density of the BP, manifests itself through the variations in the radiation transfer response in the heterogeneous 2D material.

**Fig. 4:**
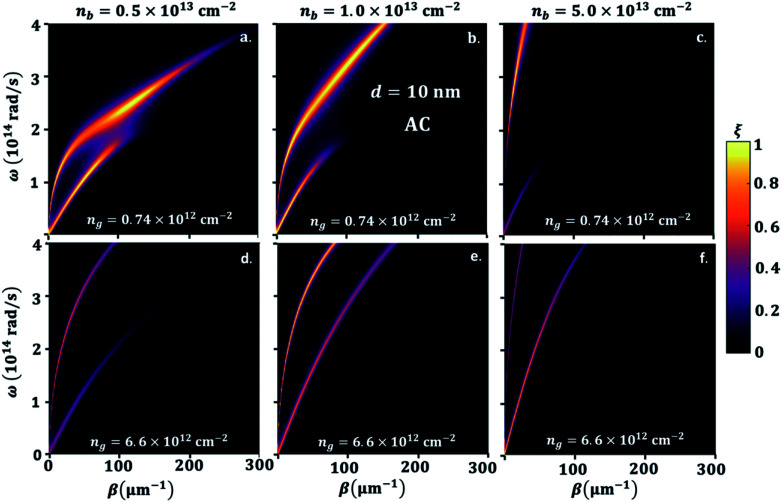
Color map of photon transmission probability (*ξ*) between suspended BP emitter and graphene sheet receiver (G), and black phosphorene sheet (B) emitter arm chair (AC) direction along *x*-axis orientation separation distance, *d* = 10 nm, electron density of graphene, *n*_g_ = 0.74 × 10^12^ cm^−2^, and BP, *n*_b_, (a) 5.0 × 10^12^ cm^−2^, (b) 1.0 × 10^13^ cm^−2^, (c) 5.0 × 10^13^ cm^−2^, respectively and *n*_g_ = 6.6 × 10^12^ cm^−2^, and BP, *n*_b_, (d) 5.0 × 10^12^ cm^−2^, (e) 1.0 × 10^13^ cm^−2^, (f) 5.0 × 10^13^ cm^−2^, respectively.

To explain the different phenomena observed in Fig. 2 and 3, we consider the heat transmission coefficient *ξ*(*ω*,*β*) in eqn (8), between BP and GR heterogenous system. Fig. 4 presents a contour plot of the evolution of the heat transition/photon transmission probability, or energy transmission coefficient distributions *ξ*(*ω*,*β*) of the BPE–GRR system, similar to the case in Fig. 2. These transmission coefficient results are for *n*_b_ = 0.5 × 10^13^ cm^−2^, 1.0 × 10^13^ cm^−2^ and 5.0 × 10^13^ cm^−2^ and *n*_g_ = 0.74 × 10^12^ cm^−2^ and 6.6 × 10^12^ cm^−2^ at separation distance, *d* = 10 nm.

The contours show two bright bands covering a wide range of frequencies and momentum. These bright bands correspond to the symmetric (lower frequencies) and anti-symmetric (higher frequencies) branches of the coupled SPPs between 2D materials.^27,28^ Here one mode is originated from the BPE side and a second from the GRR; they are labelled SPB and SPG, respectively. The band are characteristics reflections of the SPPs dispersion band and an indication of electromagnetic coupling process of BP and graphene. The strength of the two bright band colors indicates a high transmission coefficient and the coupling strength of the SPPs of black phosphorene and graphene. Therefore, they are the major contributors to the SHF between black phosphorene and graphene. In Fig. 4a, we observe strong nearly overlapping and small separation of the bright band transition probability for *n*_b_ = 0.5 × 10^13^ cm^−2^ and for *n*_g_ = 0.74 × 10^12^ cm^−2^. The upper branch is BP like, and lower branch is graphene like. Short momentum and frequency spanning in the lower branch are due to the interband transition damping, specifically at energies *ħω* < 2*μ*.^26,28,34^

However, strongly overlapping bright bands are produced for low doping BP and graphene, resulting in overall a higher NFRHT, Fig. 3a. The two resonances branches show a progressive shift and move further apart, especially for a large *n*_b_, indicating mismatch between the hybrid SPPs modes of the BP emitter and the graphene receiver, Fig. 4b and c. The motions toward a lower wave vector result in a decrease in transmission and the NFRHT, Fig. 2b and c, due to weak coupling. In case of *n*_g_ = 6.6 × 10^12^ cm^−2^, Fig. 4d–f, both branches cover a large frequency range as the interband damping effect of graphene weakens. However, progressive increase of *n*_b_ similarly increases movement of the bright band and shifts towards higher *ω* and a lower *β*. The rise in strong offsets of the branches lead to the overall reduction of the NFRHT observed at a large ng (Fig. 2b–c) and ESI Fig. 3.†

**Fig. 5:**
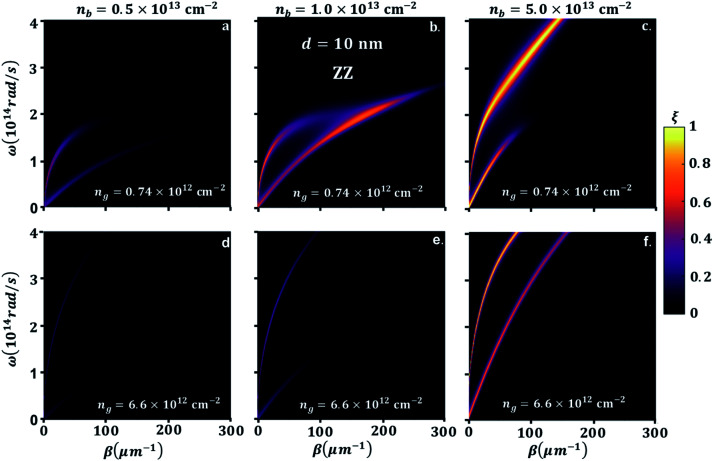
Color map of photon transmission probability (*ξ*) between suspended BP emitter and graphene sheet receiver (G), and black phosphorene sheet (B) receiver zigzag (ZZ) direction along *x*-axis orientation separation distance, *d* = 10 nm, electron density of graphene, *n*_g_ = 0.74 × 10^12^ cm^−2^, and BP, *n*_b_, (a) 5.0 × 10^13^ cm^−2^, (b) 1.0 × 10^13^ cm^−2^, (c) 5.0 × 10^13^ cm^−2^, respectively and *n*_g_ = 6.6 × 10^12^ cm^−2^, and BP, *n*_b_, (d) 5.0 × 10^13^ cm^−2^, (e) 1.0 × 10^13^ cm^−2^, (f) 5.0 × 10^13^ cm^−2^, respectively.

In order to understand the significance of anisotropy effects in the photon transmission and near-field radiation heat transfer, we look in detail at Fig. 5, where BP ZZ-direction is aligned in *x* and AC is aligned in the *y*-direction, having the same parameters as in Fig. 4. Similar to the hybridization effect of the surface plasmons of a BP–GR discussed above, two bright bands are observed. As expected, the effective mass difference has a strong influence on the heat transmission. On the one hand, the greater effective mass of electrons in ZZ direction results in energies of the bright bands that extends into longer momentum vector, hence extremely confined modes of SPPs. On the other hand, the momentum vector can be highly damped, spanning a smaller energy range. For example, when *n*_g_ = 0.74 × 10^12^ cm^−2^ and *n*_b_ = 5.0 × 10^12^ cm^−2^ or 1.0 × 10^13^ cm^−2^, with increasing *β*, we observe that the upper bright band and the lower bright band span lower frequency ranges (Fig. 4a and b). In this case the hybrid modes upper branch is graphene like, and lower branch is BP like. For larger *β*, this gap narrows because of the bending of the upper band as interband transitions comes into play. The maximum hybridization for *n*_g_ = 0.74 × 10^12^ cm^−2^ and *n*_b_ = 1.0 × 10^13^ cm^−2^, Fig. 4b, results in a noticeable illustration of the physical origins of higher SHF peaks observed in Fig. 3a. On the other hand, SHF decreases when *n*_b_ increases to 5.0 × 10^13^ cm^−2^. Since the top branch band that is BP like spans most of the frequency range, the lower bright band that is graphene like stays in a small frequency range with decreased *β*. In graphene, the carrier density is *n*_g_ = 6.6 × 10^12^ cm^−2^, Fig. 5c–e, top branch extends covering most of the frequency range, so interband transitions of graphene are negligible. The lower branch covers a small frequency range with strong confinement for *n*_b_ = 5.0 × 10^12^ cm^−2^ and 1.0 × 10^13^ cm^−2^ moving to close to GR branch. The bright modes supported by BP for *n*_b_ = 5.0 × 10^13^ cm^−2^ move closer to GR and the heat transmission increases significantly. Although the bands do not reach a point of overlap, in contrast to lower number density, there is an indication for the origin of maximum NFRHT in Fig. 4c. This also consistent for *n*_g_ = 2.65 × 10^13^ cm^−2^ as shown in ESI Fig. 3.† The large range of *ω* covered in the heat transmission factor of the AC direction in BP clearly demonstrate the broad NFRHT as compared to ZZ oriented BP and preferable enhancement in the heterogeneous 2D, this is also further supported in the transmission probability description for highly doped graphene, see ESI Fig. 3.†

### Distance effect

The strong modulation of the NFRHT between GR and BP is shown to be quite generic, and it appears to be influenced by a wide range of parameters. We test this concept by exploring the total radiation, *Q*, computed from eqn (7) as a function of *μ*, *n* and the gap size, *d*. We quantify the various parameters significance, first by defining the total heat radiation heat transfer flux ratio, *η* = *Q*(*n*_g_,*n*_b_)_GB_/*Q*(*n*_g_,*n*_g_)_GG_, as an efficiency factor to gain better insight into the performance of BP and graphene, where *Q*(*n*_g_,*n*_b_)_GB_ is the BPE–GRR system total heat flux and *Q*(*n*_g_,*n*_g_)_GG_ is the GRE–GRR system total heat flux.

**Fig. 6:**
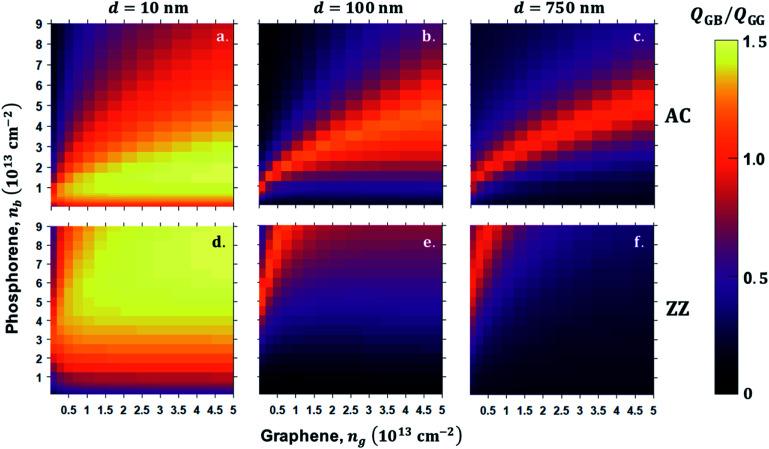
Shows the heat flux ratio between graphene sheet-BP sheet *Q*_GB_ and graphene–graphene sheet *Q*_GG_ for carrier density of graphene 0.74 × 10^12^ cm^−2^ to 5.0 × 10^13^ cm^−2^*versus* BP carrier density (*n*_b_) 2.0 × 10^12^ cm^−2^ to 9.0 × 10^13^ cm^−2^ vacuum gap distance *d* 10 nm, 100 nm and 750 nm black phosphorene sheet, receiver AC direction along *x*-axis orientation for (a, b and c) and ZZ direction along *x*-axis orientation for d, e and f.

The total heat flux, *Q*, is computed for a range of BP and graphene carrier densities for extremely narrow separation *d* = 10 nm, intermediate *d* = 100 nm, and a larger separation *d* = 750 nm, which are displayed in Fig. 6a–g for AC orientation and ratio, ZZ orientation system, and graphene–graphene system. As the vacuum gap distance increases from *d* = 10 nm to *d* = 500 and 750 nm, the monotonic decrease of both maximum and minimum total heat flux is obvious because of the weaker SPPs coupling at a larger gap distance. In addition, this reduction is a result of weak near-field coupling and short propagation distance of the evanescence field, which decreases the available of plasmonically-activated modes. The heat flux ratio reaches up to the maximum scale 1.5, at *d* = 10 nm in both AC and ZZ orientation, Fig. 6a and d, respectively. More importantly, *η*, diminishes for *n*_b_ of BP and for all *n*_g_ in AC orientation, while in ZZ orientation of BP leads to a greater *η*. From this theoretical analysis, we can conclude that BP–GR system shows superiorities compared to GR–GR in designing super Plank radiation heat transfer. As the separation increases further, efficiency of *η* > 1 is still sustained for the selected doping range, Fig. 6b, c, e and f. For *n*_g_ from 0.74 × 10^12^ cm^−2^ to 4.7 × 10^13^ cm^−2^ and *n*_b_ from 2.0 × 10^12^ cm^−2^ to 9.0 × 10^13^ cm^−2^, there is a linear bright region that shows efficiency *η* > 1. Specifically, for *n*_g_ < 1.18 × 10^13^ cm^−2^ and *n*_b_ above 2.0 × 10^12^ cm^−2^ in ZZ orientation and from 0.1 eV to 0.8 eV and BP doping level up to *n*_b_ = 5.0 × 10^12^ cm^−2^ AC orientation. Away from this BP and graphene carrier density range, the ratio is less than 1 irrespective of the BP orientation, which is an undesirable case for NFRHT enhancement of the heterogeneous 2D BP–GR system. However, this theoretical result suggests an alternate solution of realizing enhanced and reduced near-field radiation heat transfer mechanism. In particular, we can freely control the functionality of this heterogeneous system enhancing and reducing heat transfer by changing the orientation of BP and doping of both BP and graphene. Furthermore, our results demonstrate the superiority of this heterogeneous 2D material pair, which give rise to the anisotropic thermal emission by physical reorientation of BP in addition to gate-modulation of graphene and doping of BP.

## Conclusions

Our work explores NFRHT originated from SPPs assisted photon transmission for a pair coupled graphene and BP heterogeneous 2D materials. We found that the radiation beyond the Plank limit and enhanced photon tunnelling is favourable in a homogenous graphene–graphene system for low-gate modulation (<3.0 × 10^12^ cm^−2^). However, in the BPE–GRR system, the NFRHT critical value is significantly enhanced when a gate (>3.0 × 10^12^ cm^−2^) is applied to graphene and BP for selective doping. By choosing appropriate parameters of doping level of BP and graphene, a NFRHT 4 times higher can be achieved in BPE–GRR than GRE–GRR. This enhanced NFRHT effect is also realized in the total heat flux BP–GR pairs leading to more than 5 times higher than GR–GR system at *d* < 100 nm. In addition, by varying the relative orientation of the BP layer, one can switch from amplified NFRHT to reduced or *vice versa*. The NFRHT in 2D heterogeneous system resulting from preferential orientation would be dependent on the combination of gate-voltage (carrier density). Thus, for a given carrier density of BP and graphene, the heat tunnelling should be adjusted to obtain the desired radiation transfer either enhanced or reduced. We believe that our work contributes to the active pursuit in the field of SPPs supporting hybrid nonidentical 2D structures both made from atomically thin (few layers) materials. This study can further motivate future studies of hybrid surface plasmon induced NFRHT between new discovered anisotropic 2D ReS_2_ and ReSe_2_.^57,58^

## Conflicts of interest

The authors declare no conflict of interest.

## Supplementary Material

RA-009-C9RA04695J-s001
