# Supplementary material for: Gate voltage and doping effects on near-field radiation heat transfer in plasmonic heterogeneous pairs of graphene and black phosphorene
Source: RSC Adv. 2019 Sep 17;9(50):29173–81. doi: 10.1039/c9ra04695j (PMC9071808; doi:10.1039/c9ra04695j)
Supplement: RA-009-C9RA04695J-s001 [file RA-009-C9RA04695J-s001.pdf]

Electronic Supplementary Material (ESI) for RSC Advances.
This journal is © The Royal Society of Chemistry 2019

Supplementary Material

for:

Gate Voltage and Doping effect on Near-Field Radiation Heat Transfer in Plasmonics Heterogeneous Pairs of Graphene and Black Phosphorene

Desalegn T. Debu,*[a] M. Hasan Doha,[a] Hugh O. H. Churchill,[a,b] and Joseph. B. Herzog,[c]

[a] University of Arkansas, Department of Physics, Fayetteville, Arkansas 72701, USA.
[b] Institute of Nanoscale Science and Engineering, University of Arkansas, Fayetteville, Arkansas 72701, USA.
[c] University of Indianapolis, R. B. Annis School of Engineering, Indianapolis 46227, USA.

S1. Near field radiation heat between graphene emitter and graphene receiver

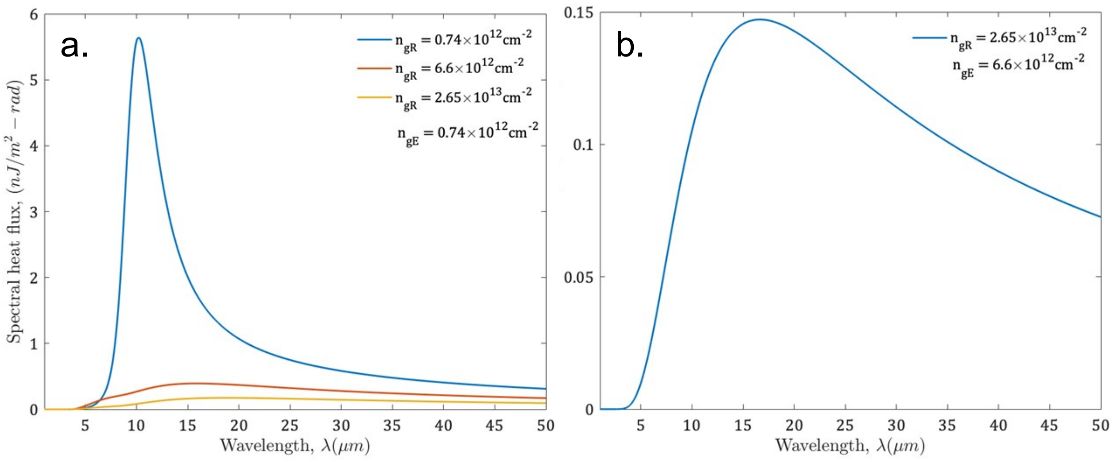

Figure S1: Spectral heat flux comparison between the similar optical property of graphene and different optical property of graphene (a) low doping level (chemical potential) of emitter graphene is set to which is n_{gE} = $0.74 \times 10^{12} cm^{-2}$ and n_{gR} = $0.74 \times 10^{12} cm^{-2}$(blue), n_{gR} = $6.6 \times 10^{12} cm^{-2}$(brown) and n_{gR} = $2.65 \times 10^{12} cm^{-2}$(yellow). As the receiver doping level of the graphene increases the heat transfer between graphene-graphene decreases. This is also shown in (b) for n_{gE} $6.6 \times 10^{12} cm^{-2}$and n_{gR} = $2.65 \times 10^{13} cm^{-2}$(blue).

S2. Near Field Radiation Heat Transfer between black phosphorene emitter and receiver

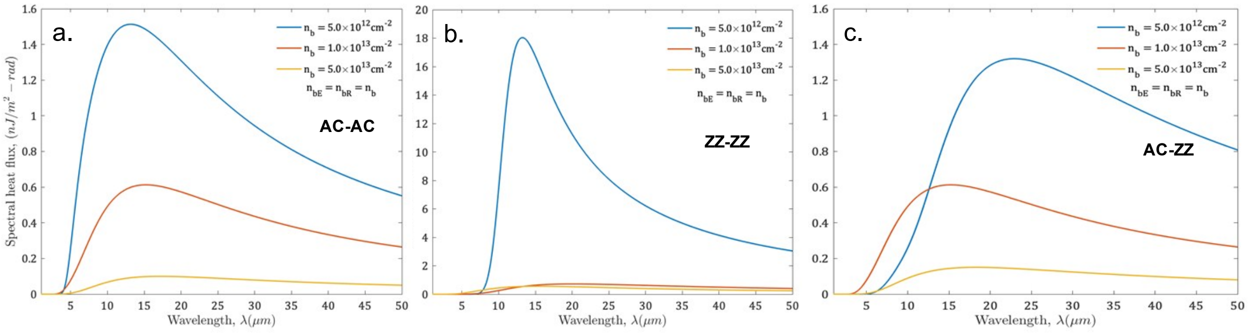

Figure S2: Spectral heat flux comparison between the black phosphorene pairs with n_{bE} = n_{bR} = n_b where $5.0 \times 10^{12} cm^{-2}$(blue), $1.0 \times 10^{13} cm^{-2}$(brown), $5.0 \times 10^{13} cm^{-2}$(yellow), (a) armchair emitter -armchair receiver, (b) zigzag emitter - zigzag receiver and (c) armchair emitter - zigzag receiver orientation.

In the case of NFRHT between identical optical properties of black phosphorene pairs, we employ a three-carrier density used in Figure S2 and S3. The SHF of AC-AC, ZZ-ZZ, and AC-ZZ orientation of emitter and receiver shows significant variation. Compared with the NFRHT for GRE-GRR, and BPE-GRR, the SHF between identical emitter and receiver of black phosphorene pair in ZZ orientation shows better performance.

S3. Heat tunneling probability between high chemical potential graphene and black phosphorene

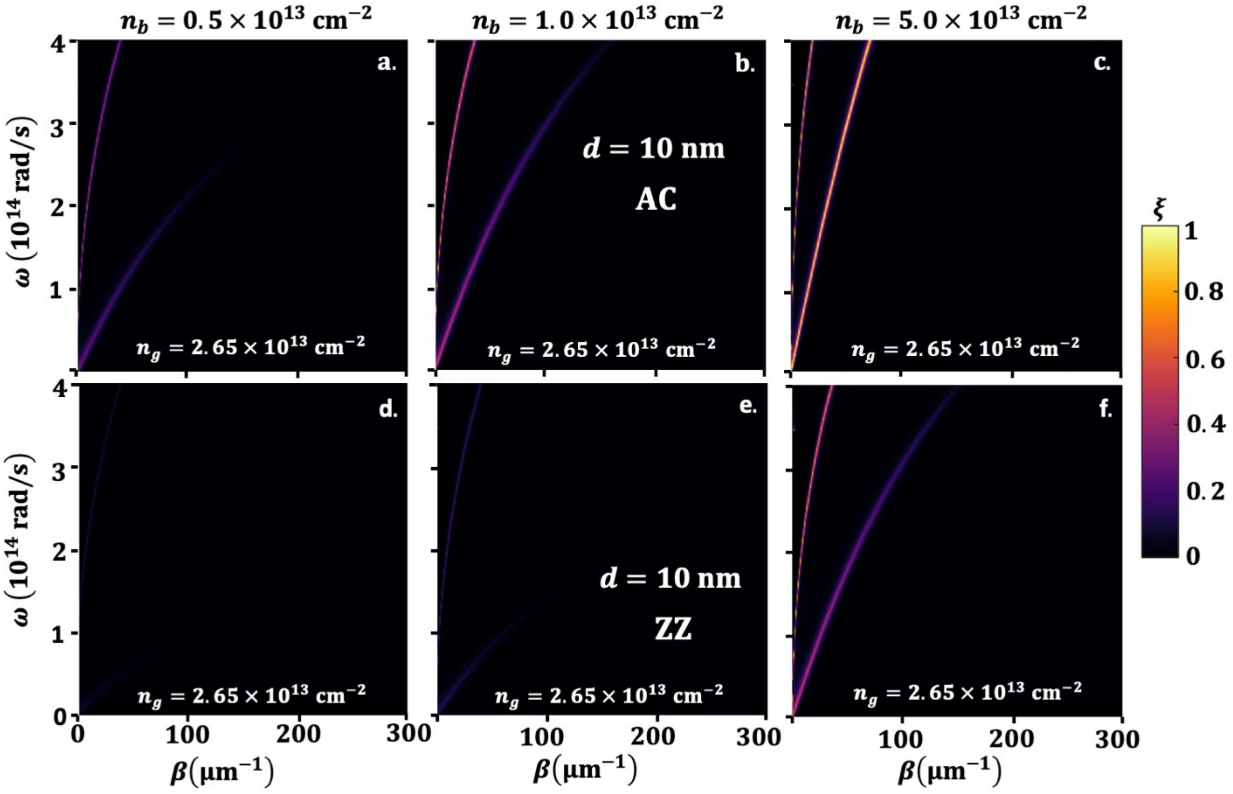

Figure S3: Color map of photon transmission probability (ξ) between suspended BP emitter and graphene sheet receiver (G), and black phosphorene sheet (B) emitter armchair (AC) direction along x-axis orientation separation distance, d = 10 nm, electron density of graphene, n_g = $2.65 \times 10^{13} cm^{-2}$, and BP AC direction, n_b, (a) $5.0 \times 10^{12} cm^{-2}$, (b) $1.0 \times 10^{13} cm^{-2}$, (c) $5.0 \times 10^{13} cm^{-2}$, respectively and along the ZZ direction, n_b, (d) $5.0 \times 10^{12} cm^{-2}$, (e) $1.0 \times 10^{13} cm^{-2}$, (f) $5.0 \times 10^{13} cm^{-2}$, respectively.

From the contour plot of photon tunneling probability, the bright bands are further apart in ZZ direction at low BP carrier density. The branches move closer with shorter momentum as the doping of BP increases providing increased SHF. The rise in chemical potential result in strong offsets of the branches lead to the overall reduction of the NFRHT observed at a large n_g (Figure 2c and Figure 3c). We observed that the enhancement become smaller as the chemical potential becomes higher in spite of similar BP doping level. Moreover, the spectra heat flux, depending on the BP carrier density, can be enhanced or reduced. This result is consistent with the result discussion we provided in the original manuscript.
